# Personalised exercise rehabilitation in cancer survivorship: the percs triage and referral system study protocol

**DOI:** 10.1186/s12885-024-12266-x

**Published:** 2024-04-23

**Authors:** Louise Brennan, Grainne Sheill, Sonya Collier, Peter Browne, Claire L. Donohoe, Linda O’Neill, Juliette Hussey, Emer M. Guinan

**Affiliations:** 1 Department of Surgery, School of Medicine, Trinity College Dublin, Dublin, Ireland; 2 Trinity St James’s Cancer Institute, Dublin, Ireland; 3 Department of Physiotherapy, St James’s Hospital, Dublin, Ireland; 4 Psycho-Oncology Unit, St. James’s Hospital, Dublin, Ireland; 5 Trinity St James’s Cancer Institute Patient Representative Group, Trinity St James’s Cancer Institute, Dublin, Ireland; 6 Department of Surgery, St James’s Hospital, Dublin, Ireland; 7 Discipline of Physiotherapy, School of Medicine, Trinity College Dublin, Dublin, Ireland; 8 Wellcome-Health Research Board Clinical Research Facility, Trinity College, St James’s Hospital, Dublin, Ireland

**Keywords:** Cancer, Survivorship, Rehabilitation, Exercise, Triage, Implementation, Function, Psychosocial, COVID-19, Physiotherapy

## Abstract

**Background:**

To effectively embed exercise rehabilitation in cancer survivorship care, a co-ordinated system of acute and community exercise rehabilitation services, forming a stepped model of care, is recommended. Patients can be directed to the exercise rehabilitation service which best meets their needs through a system of assessment, triage and referral. Triage and referral systems are not yet widely applied in cancer survivorship practice and need to be evaluated in real-world contexts. The PERCS (Personalised Exercise Rehabilitation in Cancer Survivorship) study aims to evaluate the real-world application of an exercise rehabilitation triage and referral system in cancer survivors treated during the COVID-19 pandemic. Secondary aims are to evaluate change in physical and psychosocial outcomes, and to qualitatively evaluate the impact of the system and patient experiences, at three months after application of the triage and referral system.

**Methods:**

This study will assess the implementation of an exercise rehabilitation triage and referral system within the context of a physiotherapy-led cancer rehabilitation clinic for cancer survivors who received cancer treatment during the COVID-19 pandemic. The PERCS triage and referral system supports decision making in exercise rehabilitation referral by recommending one of three pathways: independent exercise; fitness professional referral; or health professional referral. Up to 100 adult cancer survivors treated during the COVID-19 pandemic who have completed treatment and have no signs of active disease will be recruited. We will assess participants’ physical and psychosocial wellbeing and evaluate whether medical clearance for exercise is needed. Participants will then be triaged to a referral pathway and an exercise recommendation will be collaboratively decided. Reassessment will be after 12 weeks. Primary outcomes are implementation-related, guided by the RE-AIM framework. Secondary outcomes include physical function, psychosocial wellbeing and exercise levels. Qualitative analysis of semi-structured interviews guided by the Consolidated Framework for Implementation Research (CFIR) will provide insights on implementation and system impact.

**Discussion:**

The PERCS study will investigate the real-world application of a cancer rehabilitation triage and referral system. This will provide proof of concept evidence for this triage approach and important insights on the implementation of a triage system in a specialist cancer centre.

**Trial registration:**

This study is registered on ClinicalTrials.gov, registration number: NCT05615285, date registered: 21st October 2022.

**Supplementary Information:**

The online version contains supplementary material available at 10.1186/s12885-024-12266-x.

## Background

The immediate and long-term sequelae of cancer treatment, including cardiopulmonary deconditioning, impaired bone health, altered body composition, and increased rates of fatigue, depression and anxiety is well-documented [1, 2]. Compelling evidence from randomised clinical trials shows aerobic and resistance exercise training can have positive impact across numerous physical and psychosocial outcomes including anxiety, depression, cancer-related fatigue, health-related quality of life (QOL) and self-reported physical functioning [3]. This evidence was synthesised in the 2019 American College of Sports Medicine (ACSM) Exercise Guidelines for Cancer Survivors, which presented the minimal recommended levels of exercise for cancer survivors as: 30 min of moderate intensity aerobic exercise three times per week and twice-weekly resistance training [3]. These guidelines support exercise prescription for all people living with and after cancer.

Internationally, expert groups have called for better integration of exercise rehabilitation into cancer care pathways [4, 5]. To effectively translate the ACSM Exercise Guidelines research into practice, the development of a collaborative, coordinated system between hospital-based and community-based programmes, with established referral pathways enabling access to rehabilitative care from diagnosis to the post-treatment period is recommended. This should be underpinned by a stepped model of care that directs patients to the right level of service to meet their individual needs [6]. Currently, this model of care remains aspirational in most healthcare systems [6]. Research by our group finds that, in Ireland, people with and after cancer frequently do not receive support to enable them to become more physically active due to under-resourced and disjointed acute and community services, and a lack of information and awareness by both patients and the oncology multi-disciplinary team [7].

People living with and after cancer need a range of exercise rehabilitation services, and the level of professional support or supervision required depends on factors such as level of impairment, co-morbidities, and exercise-related self-efficacy. Within a stepped model of care, exercise rehabilitation can be categorised into different ‘levels’, which are typically classified as i) unsupervised, unspecialised exercise rehabilitation; ii). supervised, community-based exercise rehabilitation, broadly specialised to those with a history of cancer or chronic disease; iii). highly specialised exercise rehabilitation, supervised by a specialist healthcare professional [8, 9]. A screening approach which triages people according to their level of need and directly refers to the most suitable exercise rehabilitation service can contribute to a patient-centred, efficient pathway of care, and can prioritise specialist services for those who most need them [10].

There are exciting ongoing efforts internationally to develop stepped-care models and triage and referral systems for exercise rehabilitation in cancer survivorship. The Cancer Rehabilitation to Recreation (CaReR) Framework [11] aims to increase physical activity after cancer through a three-phase framework spanning Rehabilitation, Fitness and Recreation. The framework does not include a triage system to support decision making in identifying which phase of the framework best meets a patient’s needs. The theoretical model, which is proposed as an amalgamation of the Stepped Care Framework and the Transformative Exercise Framework [8, 11, 12], incorporates different levels of clinical oversight, from fitness professional to physiotherapist, along a spectrum of physical activity counselling across hospital and community settings. Fitness professionals can play an important and impactful role in exercise rehabilitation of cancer survivors with lower levels of impairment, by delivering exercise programmes which are generally community-based and more accessible and affordable than programmes in healthcare settings [5]. Rehabilitation programmes, delivered by allied health professionals including physiotherapist, clinical exercise physiologists or occupational therapists, are most suitable for patients with cancer-related comorbidities or physical impairments [5]. The context for this paper is a physiotherapy-led clinic. Physiotherapists are core members of the oncology multi-disciplinary team who specialise in rehabilitation and exercise prescription [7], and can assess and manage many of the common physical side effects of cancer treatment such as weakness, cachexia, fatigue, loss of balance, lymphoedema and pain [13]. In a recent qualitative analysis of semi-structured interviews with oncology-specialist physiotherapists in Ireland, physiotherapists called for all cancer survivors to have access to a physiotherapy assessment and for greater emphasis on improving QOL within cancer care but reported a lack of funding and resources to address patient needs [7].

The Exercise in Cancer Evaluation and Decision Support (EXCEEDS) triage model is a two-part decision support tool designed to be used at the point of care by multi-disciplinary users. EXCEEDS evaluates cancer survivors’ physical activity levels and chronic disease risks and recommends an appropriate level of exercise service: cancer rehabilitation, clinically supervised exercise, supervised community-based cancer specific exercise or unsupervised, generic community-based exercise [9]. The EXCEEDS algorithm has not yet been evaluated in practice and the triage outcomes may be difficult to implement in Ireland, where there is limited provision of supervised exercise programmes for cancer survivors in community- and healthcare-based settings (both public and private) [14]. This situation is not dissimilar to the situation in many countries where exercise rehabilitation services for cancer survivors are only starting to develop [15, 16]. There is a need for an exercise rehabilitation triage and referral system which will work in a country with limited cancer rehabilitation resources [7].

To be effectively implemented, triage and referral systems should be easily and rapidly applicable in practice, should be adaptable to different clinical contexts, and should optimally utilise the services available in the local healthcare setting [17, 18]. In line with the best available evidence in exercise oncology, such a system should be based on the 2019 ACSM guidelines for exercise in patients in cancer, and should consider patients’ current levels of exercise (both pre- and post-cancer treatment) while also being sensitive to identify possible individual risks of increasing exercise [19].

In June 2021, the Irish Cancer Society, in partnership with the National Cancer Control Programme in Ireland, launched the COVID-Cancer Rapid Response Award. The aim was to identify evidence-based mitigations for the burdens brought about by the COVID-19 pandemic on people living with or beyond cancer in Ireland. The pandemic had profound physical and psychosocial effects on the general population and there are major concerns that cancer survivors may be disproportionally impacted [20]. Internationally, there was a high prevalence of psychological strain, stress and isolation among cancer survivors during this period [21]. In Northern Ireland, 61% of previously active cancer survivors, reported a psychosocial impact of the COVID-19 restrictions including loneliness, lack of social support, decreased motivation to exercise, fear, anxiety and depression, while 32% reported a physical impact including deterioration in health and fitness, increased pain and body weight, and changes in dietary habits habits [22]. Alongside the existing need to make evidence-based exercise rehabilitation a priority for cancer care, there is now an additional, urgent need to mitigate the dual impact of cancer treatment and COVID-19 on physical and psychosocial health.

The Personalised Exercise Rehabilitation in Cancer Survivorship (PERCS) study, which is funded through the COVID-Cancer Rapid Response Award, will test the real-world application of an exercise rehabilitation triage and referral system within a physiotherapy-led cancer rehabilitation clinic. The system aims to improve exercise levels in participants by optimally utilising existing exercise rehabilitation services, particularly those in the community, while reserving specialist exercise rehabilitation services delivered by physiotherapists for patients who require a more specialised level of care. The triage and referral system will be applied with people who received cancer treatment during the height of the COVID-19 pandemic, with a view to providing additional supports to this group who experienced exceptional challenges during their treatment.

## Methods

### Study aims

The overall aim of this study is to evaluate the real-world application of an exercise rehabilitation triage and referral system on physical and psychosocial outcomes in cancer survivors treated during the COVID-19 pandemic. The triage and referral system will be delivered within the context of a physiotherapy-led cancer rehabilitation clinic in a specialist national cancer centre.

The study objectives are to:

1. Apply an exercise rehabilitation triage and referral system for cancer survivors in a single specialist cancer centre.
2. Describe the real-world implementation of the triage and referral system.
3. Evaluate change in real-world physical functioning outcomes and psychosocial outcomes after 12 weeks following application of the triage and referral system.
4. Qualitatively evaluate impact on health-related QOL, patient experience and psychosocial outcomes after 12 weeks following application of the triage and referral system.

### Study design

**Fig. 1:**
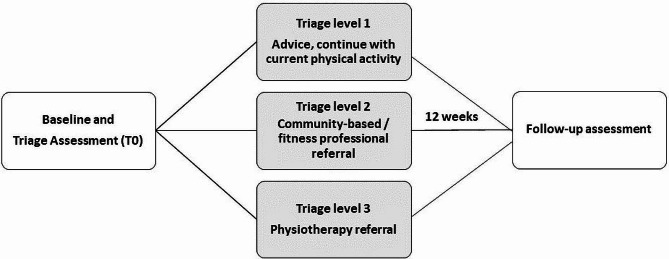
Overview of study design

This study will use a pre-post-test design to examine the real-world application of an exercise rehabilitation triage and referral system for cancer survivors. The exercise rehabilitation triage and referral system will be applied following a baseline assessment (T0) and will guide patient referral to one of three exercise rehabilitation pathways (Fig. 1). A follow-up assessment will be completed after 12-weeks (T1). The study will take place in the Wellcome-Health Research Board (HRB) Clinical Research Facility (CRF) at St James’s Hospital, Dublin. Ethical approval has been obtained from the Tallaght University Hospital/ St James’s Hospital Joint Research Ethics Committee (Study ID 0670) and hospital approval to conduct research from the Research and Innovation Office at St James’s Hospital. Any amendment to the protocol which may impact on the conduct of the study will be submitted as an amendment for approval to the ethics committee. PERCS study is registered with ClinicalTrials.gov (trial identifier NCT05615285) and will be performed according to the Declaration of Helsinki.

### Study participants

PERCS will recruit up to 100 patients who have been diagnosed with cancer at St James’s Hospital between March 2020 and March 2022. St James’s Hospital is the largest cancer centre in Ireland with high-volume national, supra-regional and regional teams and structures for multiple malignancies and dedicated cancer prehabilitation and rehabilitation pathways managed by a team of clinical specialist physiotherapists.

Participants must meet the following eligibility criteria: have completed adjuvant chemotherapy and/or radiotherapy; be at least 6 weeks post-surgery; do not show signs of recurrent or metastatic disease at the time of enrolment; be over the age of 18 years; be able to provide written informed consent.

Clearance to participate in exercise, as determined by the ACSM preparticipation health screening recommendations, will be a pre-requisite for undergoing the exercise rehabilitation triage and referral system [19]. Clearance from a general practitioner or consultant to participate in the study will be required if initial assessment or study pre-screening identifies any of the following conditions: known and symptomatic cardiovascular, metabolic, or renal disease; signs or symptoms suggestive of cardiovascular, metabolic, or renal disease; and recognised precautions for exercise [19, 23].

PERCS opened for recruitment from January 2023 to June 2023 with final data collection in September 2023.

### Screening and recruitment

Potential participants will be identified by the clinical cancer prehabilitation and rehabilitation physiotherapy team from a database of patients who were referred to the OpFit Cancer Prehabilitation programme at St James’s Hospital, beginning from March 2020. Further eligibility screening will be completed by the research team. Potential participants will be sent a letter of invitation to participate, which will include a participant information leaflet (PIL) and a cover letter including contact details for the study team. One week after the anticipated arrival date of this letter, a member of the research team will follow up with a phone call to answer any questions, to confirm if the person is or is not interested in participating in the study, and, where applicable, obtain verbal consent to participate and schedule an initial assessment. Full written consent in duplicate will be obtained at the T0 assessment, after which an entry will be made on the participant’s electronic patient record that they are participating in the study and their General Practitioner will be informed by letter. Individuals who decline participation will have the opportunity to consent to providing information on their demographics, exercise levels, reasons for declining, and equality, diversity and inclusion metrics to help the research team understand the population who did not participate. A separate written informed consent form will be completed for this data collection.

### PERCS exercise rehabilitation triage and referral system

**Fig. 2:**
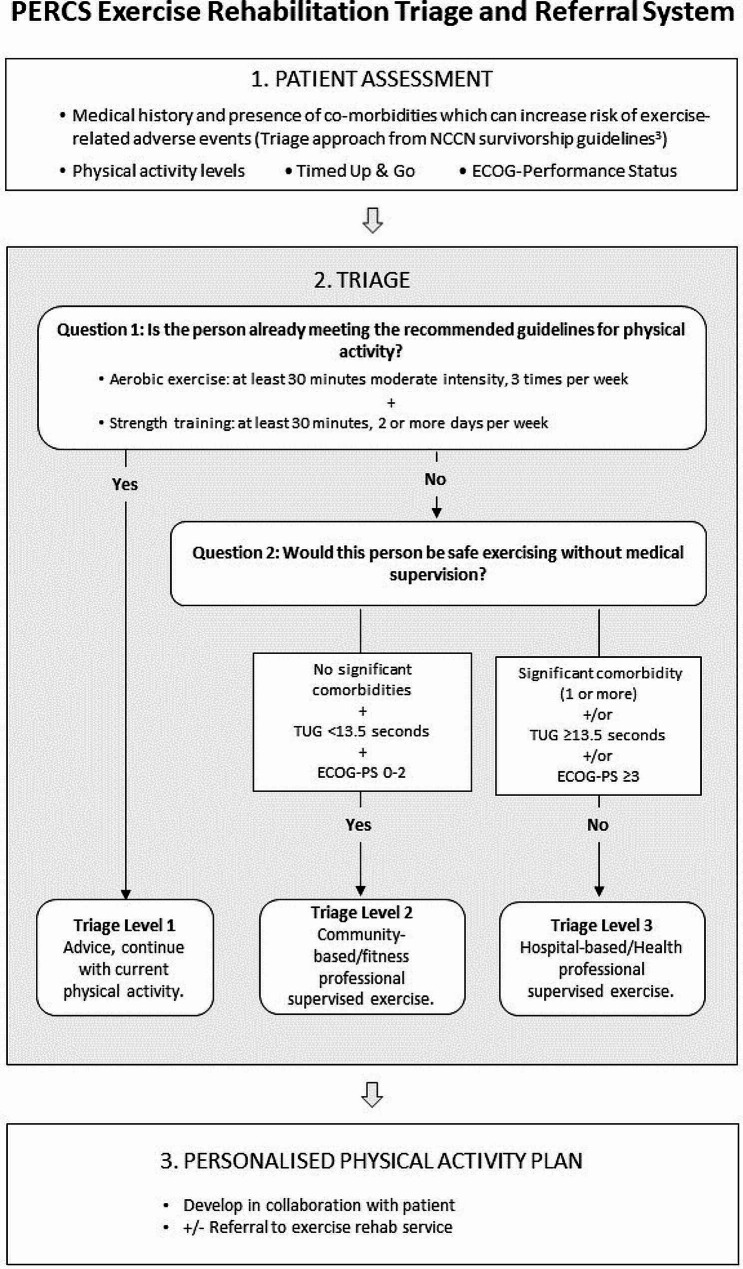
PERCS triage and referral system

The PERCS exercise rehabilitation triage and referral system was developed by the PERCS research team and an overview is presented in Fig. 2. The system involves:

1. Gathering specific information through patient assessment.
2. Completing triage questions to inform a decision on the best level of exercise rehabilitation for a patient.
3. A discussion of triage outcome with the patient, collaborative creation of an exercise plan, and making any required referrals to exercise rehabilitation services.

To complete the PERCS exercise rehabilitation triage and referral system, an assessment must be conducted to gather, at a minimum, the following information: past medical history, exercise levels, Timed Up & Go (TUG) and Eastern Cooperative Oncology Group Performance-Status (ECOG-PS). Two triage questions use assessment information to guide the user to one of three recommended pathways (‘triage levels’) for exercise rehabilitation advice and onward referral (Fig. 2) [3, 5]. The assessor will inform participants of their triage level, and a plan for exercise over the coming 12 weeks will be developed in collaboration with the participant, including making a referral as needed. In this study, the triage system will be completed again at T1 to assess for (i) triage level recategorisation and (ii) change in individual component items. Onward referral will not be performed at T1.

#### Triage question 1: exercise assessment

Question 1 identifies whether patients do or do not require additional support to become more physically active by asking if they are currently meeting the recommended levels of exercise according to the ACSM Consensus Statement for Exercise Prescription in Cancer Survivors [3]. The PERCS triage and referral system considers participants who are physically active at a moderate aerobic intensity for 30 min, 3 times per week as adherent to physical activity guidelines for aerobic exercise, and those completing at least twice weekly resistance exercise for 30 min as adherent to exercise guidelines for resistance training.

To evaluate current adherence to exercise guidelines, two questions will be asked, as recommended by Schmitz et al. (2019) [5]:

1. How many days during the past week have you performed physical activity where your heart beats faster and your breathing is harder than normal for 30 min or more?
2. How many days during the past week have you performed physical activity to increase muscle strength, such as lifting weights?

These questions are accompanied by an expert-led discussion by the physiotherapist to establish the details of frequency, intensity, type and time, and determine through clinical reasoning if the person is meeting the exercise criteria.

#### Triage question 2: assess level of supervision needed

Question 2 seeks to identify if patients require support to become more active from a healthcare professional or from a fitness professional. Participants at heightened risk for adverse events associated with increasing current exercise levels are identified by evaluation using the triage approach adapted by Campbell et al. from the National Comprehensive Cancer Network (NCCN) Survivorship Guidelines (Table 1) [3, 24], the TUG [25] and the ECOG-PS assessment of a patient’s level of functioning [26]. Increased risk of adverse events as identified by any one of these three outcomes will lead the assessor to make a referral to a health professional for a specialist level of support in becoming more physically active.

**Table 1:** Adapted National Comprehensive Cancer Network triage approach based on risk of exercise-induced adverse events [3, 24]

Description of Patients	Evaluation, prescription, and programming recommendations
No comorbidities	No further pre-exercise medical evaluation. Follow general exercise recommendations
Peripheral neuropathy, arthritis/musculoskeletal issues, poor bone health (e.g., osteopenia or osteoporosis), lymphoedema	Pre-exercise medical evaluation recommended. Modify general exercise recommendations based on assessments. Consider referral to trained personnel.
Lung or abdominal surgery, ostomy, cardiopulmonary disease, ataxia, extreme fatigue, severe nutritional deficiencies, worsening/changing physical condition (e.g., lymphoedema exacerbation), bone metastases*	Pre-exercise medical evaluation and clearance by a physician before commencing exercise. Referral to trained personnel.

*Table taken directly from the NCCN guidelines. Patients with bone metastases were not eligible to participate in this evaluation PERCS

##### Co-morbidity status

Co-morbidity status will be evaluated using NCCN triage approach based on risk of exercise-induced adverse events contained within their Survivorship Guidelines [24]. Disease-specific and treatment related side-effects which are considered to increase risk of exercise-induced adverse events will be identified in assessment using a standardised case report form (Supplemental material 1).

##### Falls risk

The Timed Up and Go test is a reliable measure of functional mobility, balance and falls risk. It records the time in seconds it takes for a participant to stands up from a chair, walk 3 m, turn back and sit in the chair. A faster time indicates better functional mobility, and a cut-off of 13.5 s is indicative of older adults at heightened falls risk [27].

##### Eastern cooperative oncology group-performance status

The ECOG-PS is a method of assessing the functional status of a patient that is widely used in oncology, particularly clinical trials, to define the population of patients to be studied in a trial, and track change in a patient’s level of functioning due to treatment during a trial (Table 2).

**Table 2:** Eastern Cooperative Oncology Group- Performance Status (ECOG-PS)

Grade	ECOG-PS
0	Fully active, able to carry on all pre-disease performance without restriction
1	Restricted in physically strenuous activity but ambulatory and able to carry out work of a light or sedentary nature, e.g., light housework, office work
2	Ambulatory and capable of all selfcare but unable to carry out any work activities; up and about more than 50% of waking hours
3	Capable of only limited selfcare; confined to bed or chair more than 50% of waking hours
4	Completely disabled; cannot carry on any selfcare; totally confined to bed or chair
5	Dead

#### Triage and referral system outcomes

The outcome of the Triage and Referral system is discussed by the physiotherapist and participant at the end of the assessment appointment. The triage and referral system leads to three possible outcomes:

##### Triage level 1

Participants who are currently meeting recommend levels of exercise will be advised by the physiotherapist to continue with their current exercise. Participants who want to increase their exercise to achieve a particular goal will receive advice but will not be referred to a service.

##### Triage level 2

Participants who are not currently exercising to recommended levels and do not require medical supervision to exercise will be referred to a ‘local exercise service’, which is led by an exercise or fitness professional who is not a regulated healthcare professional. Examples of local exercise services run by fitness professionals are: exercise programmes which are run specifically for cancer survivors, chronic disease populations or older adults; online exercise classes for chronic disease populations; a suitable local fitness class; or an exercise service provided by a community cancer support centre (charity-based).

The choice of programme will be decided in collaboration with the participant, taking into account their preference for exercise modality, service location, and other accessibility factors such as time, duration, and frequency. The PERCS physiotherapist will provide contact details of the selected programme, will make a referral where needed and will encourage self-referral where possible. To aid communication with fitness professionals, a standardised information sheet will be provided to participants outlining the results of the PERCS assessment, stating low risk of adverse events with exercise, and providing contact details for the PERCS physiotherapist.

##### Triage level 3

Participants who are not currently exercising to recommended levels and who are deemed to require medical supervision to exercise. In our context in Ireland, this is a CORU regulated health professional (https://coru.ie/), but this may vary internationally. Level 3 participants will be referred via the electronic patient record system to the Clinical Specialist Physiotherapist in Cancer Rehabilitation at St James’s Hospital (co-author GS) who will manage the referral through her clinical pathway. This may involve online or face-to-face assessment, online or face-to-face treatment sessions/exercise class sessions, discussion with the multidisciplinary team or onward referral to primary care physiotherapy services. The interventions provided by the Clinical Specialist Physiotherapist in Cancer Rehabilitation will be individualised to each participant based on clinical judgement and assessment.

All participants will be advised to visit the PERCS website (www.cancerrehabilitation.ie), which was co-designed with patient representatives at the start of the PERCS study. The aim of the website is to be a national online resource for information on exercise and cancer. The website contains concise, reliable information from trusted sources, aerobic and resistance training videos, and a national directory of exercise rehabilitation services which are suitable for people living with and after cancer. All participants will receive a follow-up telephone call one week post T0 assessment to answer any questions and support engagement in the recommended pathway. Further phone calls will be scheduled if required to support the participant with their referral pathway, e.g. to ensure referrals were received.

#### Context of implementation

For the purposes of this real-world implementation study, the exercise rehabilitation triage and referral system will be applied within the context of a physiotherapist-led ‘Cancer Rehabilitation Clinic’. In this context, the physiotherapist will apply a biopsychosocial approach to assessments, identifying holistic needs of participants, setting appropriate patient-centred goals and developing local referral pathways internally and externally to support a diverse range of rehabilitation needs.

### Measures

#### Primary outcomes: real-world implementation

PERCS primary outcomes relate to evaluating the real-world application of the Triage and Referral System, namely, an implementation analysis using RE-AIM planning and evaluation framework (Table 3) [28]. The RE-AIM framework consists of five dimensions: Reach, Effectiveness, Adoption, Implementation and Maintenance, which help to plan programmes with greater external validity, improve the chances of a programme working in a real-world setting and identify the relative strengths and weakness of an approach. Three dimensions of RE-AIM will be applied to this project: Reach, Effectiveness and Implementation. In this feasibility study, adoption or maintenance of the system will not be assessed.

**Table 3:** Implementation outcomes for PERCS study

Implementation construct	Implementation outcome	RE-AIM dimension
Rate of eligibility for recruitment	• Percentage of patients on prehabilitation list who are eligible • Percentage of referrals to the study from clinical team who are eligible • Reasons for ineligibility	Reach
Enrolment rate	• Percentage of people enrolled in study from those who were approached for recruitment. • Reasons for declining participation	Reach
Participant characteristics	• Socio-demographics, medical history and cancer history, inclusion, equality and diversity characteristics of participants. • Compare to non-participant data, where possible	Reach
Assessment attendance rates	• Percentage of participants attending scheduled assessments at T0 and T1 • Reason for non-attendance	Reach, Implementation
Attrition rates	• Percentage of participants who did or did not proceed to attend the service they were referred to • Percentage of participants who attended T0 assessment that attend T1 assessment • Reasons for non-attendance	Reach, Implementation
Engagement with referral	• Level 1 participants: percentage meeting recommended exercise levels at T1 • Level 2 participants: percentage attending local exercise programme at the agreed level of attendance; percentage meeting recommended levels of exercise at T1 • Level 3 participants: proportion of physiotherapy sessions attended per participant; percentage meeting recommended levels of exercise at T1 • Percentage meeting recommended levels of exercise on weekly basis, as per weekly diary • Change in exercise levels from T0– T1 • Exercise measured by IPAQ and assessment of adherence to ACSM guidelines	Effectiveness, Implementation
Referral outcomes	• Time from assessment to referral being sent • Percentage of referrals accepted at initial site • Time from referral sent to initial appointment	Implementation
Triage	• Percentage of people triaged to each level • Percentage triaged to another level after initial triage and why	Implementation
Safety	• Number and nature of adverse events occurring in assessment process	Implementation
Qualitative feasibility	• Feasibility data gathered from semi-structured interviews with participants	Reach, Effectiveness, Implementation

RE-AIM: Reach, Effectiveness, Adoption, Implementation, Maintenance

#### Socio-demographic data and medical history

At T0, patient demographics, medical history including cancer history and socio-economic data including equality, diversity, and inclusion (EDI) data will be collected using a standardised case report form. EDI data will be collected to meet American Society of Clinical Oncology guidance that research stakeholders should collect and publish aggregate data on diversity of trial participants [29]. The EDI data collection template was developed in line with European Union Equality data collection guidelines [30], in consultation with an external academic EDI specialist.

### Secondary outcomes

The assessment battery is presented in Table 4. Assessments will be performed at baseline (T0) and after 12-weeks (T1). The battery consists of secondary outcomes and two additional (ECOG-PS and TUG) which are used to complete the triage and referral system.

**Table 4:** Secondary outcomes of implementation study

Outcome	Measures	T0	T1
Assessment of Physical Functioning			
Exercise levels	International Physical Activity Questionnaire	X	X
	Exercise diaries		X
	Health Behaviour and Stages of Change Questionnaire Assessment questions [5]*	X X	X X
Self-Efficacy for Exercise	Self-Efficacy for Exercise Scale	X	X
Self-reported function	Patient-specific functional scale	X	X
Muscle strength	Hand Grip Strength	X	X
Functional lower body strength	30-second Sit-to-Stand	X	X
Aerobic Capacity and Endurance	Six Minute Walk Test	X	X
Anthropometrics	Height	X	X
	Weight Body Mass Index	X X	X X
Nutritional risk screening	Mini Nutritional Assessment	X	X
Assessment of Psychosocial Concerns		X	X
Quality of Life	European Organisation for Research and Treatment of Cancer Quality of Life Questionnaire	X	X
Fatigue	Multidimensional Fatigue Inventory	X	X
Anxiety and depression	Hospital Anxiety and Depression Scale	X	X
Triage assessment			
Exercise levels Comorbidity status Falls risk Functional status	Assessment questions [5]* National Comprehensive Cancer Network triage approach Timed-Up and Go Eastern Cooperative Oncology Group- Performance Status	X X X X	X X X X
Other			
Qualitative assessment	Semi–structured interviews (focus groups or 1:1)		X
Engagement with referral	Exercise diary; T1 assessment		X

*Subjective questions regarding physical activity levels are used both as a secondary outcome, and to inform the triage and referral system

#### Exercise and motivation to exercise

Exercise at T0 and T1 will be assessed using the self-administered International Physical Activity Questionnaire (IPAQ) [31]. The IPAQ (short-form) consists of 7 questions capturing activity levels during the previous 7 days. Data is processed using a standardised scoring protocol. Readiness to change will be assessed at T0 using the Health Behaviour and States of Change Questionnaire, which is underpinned by the Transtheoretical model [32] enabling categorisation of patients into precontemplation, contemplation and actions stages of change. Self-efficacy for exercise, i.e. the extent to which a person believes in their ability to execute an exercise plan [33], will be measured using the Self-Efficacy for Exercise Scale [33, 34]. This self-report scale consists of 9 statements describing adverse circumstances for exercise (e.g. ‘The weather was bothering you’) against which the user must rank their level of confidence from 0–10 that they would exercise three times per week for 20 minutes under that circumstance.

Levels of exercise will also be captured through asking the two exercise questions, described above at T0 and T1, and through completion of a weekly exercise diary capturing exercise frequency, which is tailored for each triage level. Participants assigned to Level 1 will record their weekly aerobic and strength exercise participation. Participants assigned to Level 2 will record their weekly attendance at their local exercise facility and any additional exercise completed. Participants assigned to Level 3 will record physiotherapy sessions attended and the exercise recommendations given by their physiotherapist. The weekly diary also contains contact details for the PERCS research team, information on exercise after cancer treatment and practical and safety advice for those aiming to increase their activity levels. Adherence will also be explored in semi-structured interviews and through discussion with participants at T1 assessment.

#### Hand grip strength and functional lower body strength

Hand grip strength, which provides a measure of hand and forearm strength and correlates well with overall muscle strength and physical function [35], will be measured by calibrated handheld dynamometry from a standard seated position with elbows at 90 degrees. Measurements will be taken in triplicate and the highest value recorded for data entry. Leg strength and endurance will be measured using the 30-second sit to stand test. The number of stands a person can complete from a standardised-height chair in 30 s without using arms for assistance will be recorded.

#### Self-reported function

The patient-specific functional score is a self-report outcome measure of function [36]. Participants will identify up to three important activities which they are having difficulties performing, and then rate their current ability to do each activity from 0 (unable to perform) to 10 (able to perform at the same level as before illness).

#### Anthropometric measures

Weight (kilogrammes (kg)) and height (centimetres (cm)) will be recorded by standard methods using a calibrated scales and stadiometer. Body mass index (BMI) will be calculated as weight (kg)/ height (metres (m^2^)).

#### Aerobic capacity and endurance

Aerobic capacity and endurance will be measured using the six-minute walk test, administered according to the American Thoracic Society Guidelines [37]. Participants will walk for 6 min along a 30 m walkway in a hospital corridor with the aim of achieving the furthest distance possible.

#### Nutritional risk screening

The Mini-Nutritional Assessment (MNA) is a validated nutritional screening and assessment tool that can identify malnutrition and risk of malnutrition in older populations. The MNA consists of 6 questions which can be summed to distinguish between elderly patients with: (1) adequate nutritional status, MNA > or = 24; (2) protein-calorie malnutrition, MNA < 17; (3) at risk of malnutrition, MNA between 17 and 23.5.

### Assessment of psychosocial concerns

#### Quality of life

Health-related QOL will be measured by the European Organisation for Research and Treatment of Cancer Quality of Life Questionnaire (EORTC- QLQ-C30) [38]. QOL categories include functional scales (physical, role, cognitive, emotional, social), symptom scales (fatigue, pain, and nausea and vomiting), global health status and QOL scale, in addition to several single-item symptom measures.

#### Fatigue

Fatigue will be measured using the Multidimensional Fatigue Inventory (MFI-20), a 20-item scale that measures the impact of fatigue in five dimensions: general, physical, cognitive, motivation and usual activities. It is scored from 1 to 20, with a cut-off score of ≥ 13 indicating severe fatigue [39].

#### Anxiety and depression

Anxiety and depression will be measured using the Hospital Anxiety and Depression Scale (HADS), a self-administered questionnaire and reliable instrument for detecting states of anxiety and depression in an outpatient setting (31). The HADS questionnaire has seven items each for depression and anxiety subscales. Scoring for each item ranges from zero to three, with three denoting highest anxiety or depression level. A total score of ≥ 8 points out of a possible 21 denotes considerable symptoms of anxiety or depression and a score ≥ 11 indicates a clinical case of anxiety or depression. If PERCS researchers have concerns for the psycho-social wellbeing of any participant, on the basis of these questionnaires or on other assessments findings, they will liaise with the Psycho-Oncology team in St James’s Hospital who can accept referrals or provide advice to the researchers as required.

### Qualitative assessment

At T1 assessments, a purposive sample of at least 20 participants will be invited to complete semi-structured interviews to examine their perceptions of the triage and referral system on their physical and psychosocial wellbeing, satisfaction with the system and barriers and facilitators to engagement with the system. The PERCS research team will interview participants who represent all three triage levels and mixed demographics to understand a breadth of participant experiences. The interview guide was developed using the Consolidated Framework for Implementation Research (CFIR) [40]. CFIR is an implementation framework consists of 37 constructs which are associated with successful implementation. By analysing an intervention against these constructs, the researcher can better understand the reasons why implementation was or was not successful [41]. CFIR constructs are clustered under five domains: intervention characteristics, outer setting, inner setting, characteristics of the individuals involved, and the process of implementation. PERCS researchers used the online CFIR interview guide development tool (https://cfirguide.org/guide/app/#/) to build a customised interview guide based on the CFIR constructs that are the focus of this implementation evaluation. Table 5 outlines the interview guide mapped against CFIR domains. No PERCS interview questions are mapped against the ‘process of implementation’ domain, as the four constructs in this domain (‘planning’, ‘engaging’, ‘executing’, ‘reflecting and evaluating’) are targeted to non-participant/user stakeholders, e.g. implementation leaders and opinion leaders. Interviews will be digitally audio-recorded and transcribed verbatim. Interview data will then be deductively analysed using pre-identified codes related to CFIR constructs, while also allowing for identification of new codes from the dataset [42, 43].

**Table 5:** Semi-structured interview guide, mapped against Consolidated Framework for Implementation Research (CFIR) domains and constructs

CFIR domain	CIFR construct	Interview question*
Intervention Characteristics	Evidence Strength & Quality	What are your overall thoughts about the PERCS system? What did you like/not like about PERCS?
	Relative Advantage	Are you aware of other systems/services which support people to become more active after cancer? If so, can you please describe the system/service and what you think are its advantages?
	Design Quality & Packaging	What are your thoughts on the quality of the written materials used in PERCS (PIL, diary)? Are there any changes you would recommend to these materials? Did you visit the PERCS webpage? If so, what are your thoughts on it? What did you find helpful / not helpful? Are there any changes you would recommend?
	Cost	Did it cost you anything (financially) to take part in PERCS? If so, can you describe the costs?
Outer Setting	Patient Needs & Resources	Please describe your overall experience of the PERCS service. Do you feel that the PERCS system and the PERCS team understand the needs and preferences of people who have had cancer treatment? Can you explain why? Do you think the PERCS system successfully meets the needs of people who have had cancer treatment? How?
		In general, how do you think people who have had cancer would respond to the PERCS system?
		Can you think of any reason or situation where someone would find it difficult to take part in the PERCS system? Is there any way we can make it easier for people to attend?
Inner Setting	Implementation Climate	Do you think a system such as PERCS system is needed for people after cancer? Why/why not? What did your family / partner / friends think of the PERCS system?
	Tension for Change	Are you aware of other systems/services which support people to become more active after cancer? If so, please describe. How do you think the other systems/services meet the needs of people after cancer?
Characteristics of Individuals	Self-efficacy	Before you attended your first assessment, how confident did you feel that you would be able to use the PERCS system? Why did you feel this way?
	Individual State of Change	Data obtained from Health Behaviour and Stages of Change Questionnaire at T0 & T1

*Order of questions in table relates to their relationship to CIFR dimensions; questions will be asked in an intuitive order at interview. CFIR: Consolidated Framework for Implementation Research, PERCS: Personalised Exercise Rehabilitation in Cancer Survivorship

### Data management and analysis

A data management plan will outline how research data will be handled during and after the project. The data management plan is a live document and will be reviewed regularly throughout the study. Source documents for this study will include hospital records and the study’s data collection forms. Outcome assessments will be legibly and accurately recorded in a paper-based case report form, which will be stored in a locked, secure location only accessible by PERCS researchers. Data from the case report form will then be entered into a password protected computer data repository. All participants will be allocated a unique study code. The key to the study code will be stored securely and separately to other study data. Electronic records will be stored on password protected encrypted devices. Upon completion of the study an anonymised data set will be deposited on a secure online repository in line with open access publication requirements. Direct access will be granted to authorised representatives from the host institution, CRF and regulatory authorised to permit study-related monitoring, audits, and inspections.

A descriptive analysis will be completed for relevant implementation outcomes. Summary statistics for continuous variables and categorical variables will be presented, with sub-group analysis as appropriate. Change in physical functioning and psychosocial outcomes from T0-T1 will be analysed used paired sample t-tests or the Wilcoxon test. Qualitative data will be deductively analysed using NVivo 11 (QSR International, Australia) qualitative data analysis software.

### Trial management and governance

Management of the PERCS study is overseen by a trial management group who meet biannually as standard and more frequently in response to the needs of the study. The purpose of the trial management group is to: guide study conception and development of the study protocol; review and approve protocol amendments; advise on the methodology and review any relevant new information regarding the intervention or clinical area which may impact on the running of the trial; oversee the day-to-day running of the PERCS study; and ensure that the viewpoints of all stakeholder groups are considered. Membership of the trial management group includes the principal investigator, the clinical lead, the project manager, representatives from academic and clinical physiotherapy and psycho-oncology, and three patient representatives.

### Dissemination

Findings of PERCS will be disseminated via peer-reviewed publications and conference presentations. Aggregate study results will be presented to participants and their families at an education symposium upon study completion. Anonymised data will be made available on an open access repository.

### Public and patient involvement

Three individuals from the Trinity St James’s Cancer Institute Patient Representative Group sit on the trial management group and provide input into all aspects of the study. Patient representatives were extensively involved in the co-design of the PERCS website (www.cancerrehabilitation.ie), which will be available to all participants in this current study as an additional informational resource. Through an iterative process, based in user-centred design principles, patient representatives from a range of demographic and cancer-type backgrounds guided the development of this site. The patient needs identified in this co-design process also informed the execution of this current study, e.g. the need to provide user-friendly, easy-to-read written materials (participant information leaflets, exercise diaries).

## Discussion

This study will investigate the real-world application of an exercise rehabilitation triage and referral system in cancer survivorship. The importance of triage and referral systems to support implementation of exercise rehabilitation into practice cannot be overstated. Appropriately triaging cancer survivors to the correct level of care for their needs is widely regarded as the basis of an efficient, effective stepped model of rehabilitation care [5, 8, 9].

While the PERCS triage and referral system is being pilot tested on a cohort of patients treated during the COVID-19 pandemic, the system has the potential to be applied at various point across the survivorship trajectory, and with numerous sub-populations of cancer survivors. The population and context of use will largely influence the outcomes of the triage system. For example, if applied during or soon after treatment, there would be more demand on ‘level 3’ services i.e. exercise rehabilitation with specialist healthcare professionals, as there will likely be a higher level of need in this group than in the cohort involved in this study, many of whom will be 2 years post-treatment. Other potential applications of this system include its use in people with specific cancer types, and application by other healthcare professionals, for example within a nursing-led clinic.

This study is based upon a strong evidence base showing that exercise is beneficial for both the physical and psycho-social wellbeing of cancer survivors [3]. Physical and psycho-social outcomes will be completed for all participants, allowing us to identify a more holistic impact of exercise in this sample. Participants will have the opportunity to set targeted, meaningful goals within the clinic. This important health behaviour change technique can support engagement in exercise [44], and, supplemented by use of the Patient Specific Functional Scale, can also show change in function or ability over time [36]. The physiotherapy-led clinic setting further supports the holistic management of cancer survivors, as referrals can be made to other members of the oncology multi-disciplinary team, addressing the wider needs of participants as needed.

## Conclusion

Exercise rehabilitation triage and referral systems are widely recommended to support people living with and after cancer to become more active and to improve physical and psychosocial wellbeing. To support implementation in clinical settings, real-world evaluation of these systems are needed. The PERCS triage and referral system supports decision making in exercise rehabilitation referrals and could help address issues of under-resourced specialist rehabilitation services, directing patients to best level of support for their need. The PERCS triage and referral system could be applied in a wide range of contexts and this study will explore its application in a physiotherapy-led clinic with people who were diagnosed with cancer during the COVID-19 pandemic.

### Electronic supplementary material

Below is the link to the electronic supplementary material.

Supplementary Material 1

## Data Availability

Not applicable.

## Abbreviations

ACSM: American College of Sports Medicine
BMI: Body Mass Index
Carer: Cancer Rehabilitation to Recreation
CFIR: Consolidated Framework for Implementation Research
CRF: Clinical Research Facility
ECOG-PS: Eastern Cooperative Oncology Group-Performance Status
EDI: Equality, Diversity, Inclusion
EORTC- QLQ-C30: European Organisation for Research and Treatment of Cancer Quality of Life Questionnaire
EXCEEDS: Exercise in Cancer Evaluation and Decision Support
HRB: Health Research Board
IPAQ: International Physical Activity Questionnaire
MFI-20: Multidimensional Fatigue Inventory
MNA: Mini-Nutirional Assessment
NCCN: National Comprehensive Cancer Network
PERCS: Personalised Exercise Rehabilitation in Cancer Survivorship
QOL: Quality of life
RE-AIM: Reach, Effectiveness, Adoption, Implementation, Maintenance
