# Supplementary material for: Personalised exercise rehabilitation in cancer survivorship: the percs triage and referral system study protocol
Source: BMC Cancer. 2024 Apr 23;24:517. doi: 10.1186/s12885-024-12266-x (PMC11040825; doi:10.1186/s12885-024-12266-x)
Supplement: Supplementary file 1 — Supplementary Material 1 [file 12885_2024_12266_MOESM1_ESM.pdf]

PERCS Personalised Exercise Rehabilitation in Cancer Survivorship

Case Report Form

Trial Code:

PERCS ____

Study Contact Information:

Clinical Lead SJH:

Dr Claire Donohoe, St. James's Hospital, Dublin

Principal Investigator:

Dr Emer Guinan, Assistant Professor, Dept of Clinical Medicine, Trinity College Dublin

Study Contact:

Dr Louise Brennan, Project Manager, Discipline of Surgery, Trinity Centre for Health Sciences, St. James's Hospital Dublin 8

Contact via email: percs@tcd.ie or phone: 087 090 2644

Informed Consent

Date Verbal Consent agreed	
Date Consent Form signed	

Inclusion/ Exclusion Criteria

**Inclusion Criteria (patient can only participate with strictly "yes" answers)**		
	**Yes**	**No**
Diagnosed with cancer at St James's Hospital since March 2020		
Completion of adjuvant chemotherapy and/or radiotherapy		
At least 6 weeks post-surgery		
No signs of recurrent or metastatic disease at the time of enrolment		
Over the age of 18 years		

**Exclusion Criteria (patient can only participate with strictly "no" answers)**		
	**Yes**	**No**
Presence of any absolute contraindications to exercise as per American College of Sports Medicine preparticipation health screening recommendations		
Unable to provide informed consent		

Source of referral: ☐ Consultant ☐ Cancer Specialist Nurse ☐ Physiotherapist

☐ Prehabilitation list ☐ Other (please state) _____________________

Accessibility needs identified: ☐ yes ☐ no

Please state need and actions taken to enable inclusion:
____

____

____

T0 Assessment

Date: / /2023

Participant Demographics

Age (years): _______

Gender: ☐ Female ☐ Male ☐ Other _______________

Cancer & Medical History

Cancer details:

Type

Month & year of diagnosis

Stage at diagnosis

Consultant

Treatment received:

☐ Surgery ☐ Chemotherapy ☐ Radiotherapy ☐ Hormone ☐ None ☐ Other

☐ Further hospital admissions

Details:

Cancer-related impairments<br><br>Does participant have:<br><br>☐ Peripheral neuropathy<br>☐ Arthritis/musculoskeletal issues<br>☐ Poor bone health (e.g. osteopenia or osteoporosis)<br>☐ Lymphoedema<br>☐ Bone metastases	☐ Lung or abdominal surgery<br>☐ Ostomy<br>☐ Cardiopulmonary disease<br>☐ Ataxia<br>☐ Extreme fatigue<br>☐ Severe nutritional deficiencies<br>☐ Worsening/changing physical condition<br>☐ Known haematological abnormalities
Details / Any other known complications of treatment:	

Co-morbidities *Provide details as required*

**Other cancer**	□ No	□ Yes	
**Cardiac :**	□ MI	□ Valve Surgery	□ Arrythmias
	□ CABG	□ Pacemaker	□ Hypertension
	□ Heart Failure	Other:	
**Respiratory:**	□ COPD	□ Asthma	Other:
**COVID-19**	Has participant contracted COVID-19: □ Yes □ No<br>Has participant received COVID-19 vaccine: □ Fully vaccinated □ No<br>□ Other (Details below)		
**Diabetes**	□ No	□ Yes , type 1	
	□ Yes, Type 2: oral medication		
	□ Yes, Type2: insulin dependent		
	□ Yes, type 2: diet dependent		
**Neurological**	□ No	□ Yes	
**Thyroid**	□ No	□ Yes	

**Orthopaedic**	□ Joint replacement	□ Osteoporosis	Other:
**Smoking:**	□ Never	□ Past Smoker	
	□ Current Smoker	Amount smoked daily:	
**Alcohol:**	□ No	□ Yes	Units per week:
Please provide details of any above category here:			

Current medications

Please include name and dose of all current medications

Allergies

Please specify

Symptoms and Survivorship Needs

Current symptoms

e.g. Pain, fatigue, weakness, sleep difficulties, anxiety, low mood, poor balance, sensory changes, body composition changes, breathing difficulties, swallow/ eating/ digestion difficulties, sexual health issues, incontinence, cognitive problems, other psycho-social

**Symptom**	**Timeframe**	**Other details:** severity, rx to date, identify main problem

Mobility and Function

Mobility and exercise<br>*Current mobility, aids, activities or sports*	Premorbid mobility and exercise<br>*Pre-cancer mobility, aids, activities or sports*

Falls History (past 12 months):

Social History

Family, living circumstances, work, hobbies, other responsibilities or occupations

Travel and accessibility (can be assisted by researcher calculations as needed)

Distance in Km from home to St James's Hospital (SJH):	
Time in hours & minutes from home to SJH:	
Mode of transport to SJH:	
Cost of getting to SJH:	
Any indirect costs of getting to SJH:<br>(e.g. related to childcare, work, food)	

ACSM Recommended Exercise Guidelines

Aerobic:
"How many days in the past week have you performed physical activity where your heart beats faster and your breathing is harder than normal for 30 minutes or more?"

Days per week engaged in ≥ 30 mins moderate intensity exercise: ___________
Details of exercise: ____

Resistance:
"How many days during the past week have you performed physical activity to increase muscle strength such as lifting weights?"

Days per week engaged in resistance exercise: ___________
Details of exercise: ____

Participant is meeting the recommended exercise guidelines:

☐ Yes ☐ No (Aerobic ≥ 3 days; resistance ≥ 2 days)

INTERNATIONAL PHYSICAL ACTIVITY QUESTIONNAIRE

We are interested in finding out about the kinds of physical activities that people do as part of their everyday lives. The questions will ask you about the time you spent being physically active in the **last 7 days**. Please answer each question even if you do not consider yourself to be an active person. Please think about the activities you do at work, as part of your house and yard work, to get from place to place, and in your spare time for recreation, exercise or sport.

Think about all the **vigorous** activities that you did in the **last 7 days**. **Vigorous** physical activities refer to activities that take hard physical effort and make you breathe much harder than normal. Think *only* about those physical activities that you did for at least 10 minutes at a time.

1. During the **last 7 days**, on how many days did you do **vigorous** physical activities like heavy lifting, digging, aerobics, or fast bicycling?

_____ **days per week**

☐ No vigorous physical activities → ***Skip to question 3***

2. How much time did you usually spend doing **vigorous** physical activities on one of those days?

_____ **hours per day**

_____ **minutes per day**

☐ Don't know/Not sure

Think about all the **moderate** activities that you did in the **last 7 days**. **Moderate** activities refer to activities that take moderate physical effort and make you breathe somewhat harder than normal. Think only about those physical activities that you did for at least 10 minutes at a time.

3. During the **last 7 days**, on how many days did you do **moderate** physical activities like carrying light loads, bicycling at a regular pace, or doubles tennis? Do not include walking.

_____ **days per week**

☐ No moderate physical activities → ***Skip to question 5***

4. How much time did you usually spend doing **moderate** physical activities on one of those days?

_____ **hours per day**

_____ **minutes per day**

☐ Don't know/Not sure

Think about the time you spent **walking** in the **last 7 days**. This includes at work and at home, walking to travel from place to place, and any other walking that you have done solely for recreation, sport, exercise, or leisure.

5. During the **last 7 days**, on how many days did you **walk** for at least 10 minutes at a time?

_____ **days per week**

☐ No walking → ***Skip to question 7***

6. How much time did you usually spend **walking** on one of those days?

_____ **hours per day**

_____ **minutes per day**

☐ Don't know/Not sure

The last question is about the time you spent **sitting** on weekdays during the **last 7 days**. Include time spent at work, at home, while doing course work and during leisure time. This may include time spent sitting at a desk, visiting friends, reading, or sitting or lying down to watch television.

7. During the **last 7 days**, how much time did you spend **sitting** on a **week day**?

_____ **hours per day**

_____ **minutes per day**

☐ Don't know/Not sure

This is the end of the questionnaire, thank you for participating.

Patient Specific Functional Scale

Clinician to read and fill in below: Complete at the end of the history and prior to physical examination.

Initial Assessment:

I am going to ask you to identify up to three important activities that you are unable to do or are having difficulty with as a result of your cancer diagnosis/treatment. Today, are there any activities that you are unable to do or having difficulty with because of your cancer diagnosis/ treatment? (Clinician: show scale to patient and have the patient rate each activity).

Follow-up Assessments:

When I assessed you on (state previous assessment date), you told me that you had difficulty with (read all activities from list at a time). Today, do you still have difficulty with: (read and have patient score each item in the list)?

Patient-specific activity scoring scheme (Point to one number):

0 1 2 3 4 5 6 7 8 9 10

Unable to perform activity

Able to perform activity at the same level as before diagnosis

T0 Date: / /

T1 Date: / /

**Activity**	**T0**	**T1**
1		
2		
3		
Additional		
Total		

Total score = sum of the activity scores/number of activities
Minimum detectable change (90%CI) for average score = 2 points
Minimum detectable change (90%CI) for single activity score = 3 points

PSFS developed by: Stratford, P., Gill, C., Westaway, M., & Binkley, J. (1995). Assessing disability and change on individual patients: a report of a patient specific measure. Physiotherapy Canada, 47, 258-263. Reproduced with the permission of the authors.

Mini Nutritional Assessment

Sex: ☐ Age: ☐ Weight, kg: ☐ Height, cm: ☐ Date: ☐

Complete the screen by filling in the boxes with the appropriate numbers. Total the numbers for the final screening score.

Screening

A Has food intake declined over the past 3 months due to loss of appetite, digestive problems, chewing or swallowing difficulties?
0 = severe decrease in food intake
1 = moderate decrease in food intake
2 = no decrease in food intake ☐

B Weight loss during the last 3 months
0 = weight loss greater than 3 kg (6.6 lbs)
1 = does not know
2 = weight loss between 1 and 3 kg (2.2 and 6.6 lbs)
3 = no weight loss ☐

C Mobility
0 = bed or chair bound
1 = able to get out of bed / chair but does not go out
2 = goes out ☐

D Has suffered psychological stress or acute disease in the past 3 months?
0 = yes 2 = no ☐

E Neuropsychological problems
0 = severe dementia or depression
1 = mild dementia
2 = no psychological problems ☐

F1 Body Mass Index (BMI) (weight in kg) / (height in m)^2 ☐
0 = BMI less than 19
1 = BMI 19 to less than 21
2 = BMI 21 to less than 23
3 = BMI 23 or greater ☐

IF BMI IS NOT AVAILABLE, REPLACE QUESTION F1 WITH QUESTION F2.
DO NOT ANSWER QUESTION F2 IF QUESTION F1 IS ALREADY COMPLETED.

F2 Calf circumference (CC) in cm
0 = CC less than 31
3 = CC 31 or greater ☐

Screening score
(max. 14 points) ☐☐

12-14 points: ☐ Normal nutritional status
8-11 points: ☐ At risk of malnutrition
0-7 points: ☐ Malnourished

Ref. Vellas B, Villars H, Abellan G, et al. *Overview of the MNA® - Its History and Challenges.* J Nutr Health Aging 2006;10:456-465.
Rubenstein LZ, Harker JO, Salva A, Guigoz Y, Vellas B. *Screening for Undernutrition in Geriatric Practice: Developing the Short-Form Mini Nutritional Assessment (MNA-SF).* J. Geront 2001;56A: M366-377.
Guigoz Y. *The Mini-Nutritional Assessment (MNA®) Review of the Literature - What does it tell us?* J Nutr Health Aging 2006; 10:466-487.
Kaiser MJ, Bauer JM, Ramsch C, et al. *Validation of the Mini Nutritional Assessment Short-Form (MNA®-SF): A practical tool for identification of nutritional status.* J Nutr Health Aging 2009; 13:782-788.
® Société des Produits Nestlé SA, Trademark Owners.
© Société des Produits Nestlé SA 1994, Revision 2009.

Physical Assessment

Height (cm)		Blood pressure	
Weight (kg)		Heart rate	
BMI (kg/m^2)		SpO2	

Hand Grip Strength

Left	1		Right	1		Set-up of dynamometer:<br>Document rung # 1-5
	2			2		
	3			3		

Timed Up and Go<br><br>**TUG Time:** _______ seconds	30 Second Sit to Stand<br><br>**30s STS score:** _________ stands in 30 seconds

6 Minute Walking Test

Length of course: ______m

Minute	HR	SpO2	Distance<br>*Make a mark for each length walked*
1			
2			
3			
4			
5			
6			Total distance walked:

Observations during physical assessment:

Triage Decision Making

ECOG Status

GRADE	ECOG PERFORMANCE STATUS
0	Fully active, able to carry on all pre-disease performance without restriction
1	Restricted in physically strenuous activity but ambulatory and able to carry out work of a light or sedentary nature, e.g., light house work, office work
2	Ambulatory and capable of all selfcare but unable to carry out any work activities; up and about more than 50% of waking hours
3	Capable of only limited selfcare; confined to bed or chair more than 50% of waking hours
4	Completely disabled; cannot carry on any selfcare; totally confined to bed or chair
5	Dead

Participant ECOG score: ___________

Pre-Participation Health Screening

Complete steps 1 - 3 below. 1. Clearly circle outcome of American College of Sports Medicine (2015) algorithm:

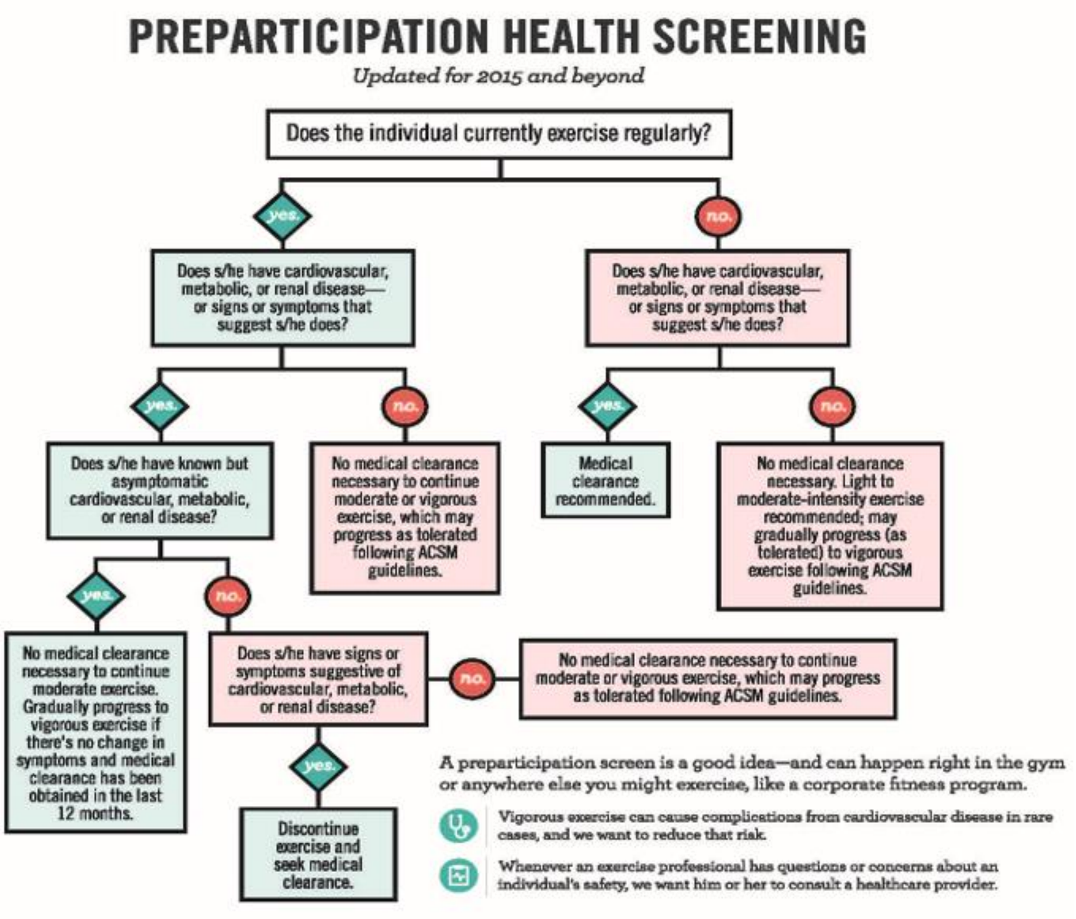

2: Tick appropriate outcome from National Comprehensive Cancer Network triage

Tick ✓	**Description of Patients**	**Evaluation, prescription, and programming recommendations**
	No comorbidities	No further pre-exercise medical evaluation.<br>Follow general exercise recommendations
	Peripheral neuropathy, arthritis/musculoskeletal issues, poor bone health (e.g. osteopenia or osteoporosis), lymphoedema	Pre-exercise medical evaluation recommended. Modify general exercise recommendations based on assessments. Consider referral to trained personnel.
	Lung or abdominal surgery, ostomy, cardiopulmonary disease, ataxia, extreme fatigue, severe nutritional deficiencies, worsening/changing physical condition (e.g. lymphoedema exacerbation), bone metastases	Pre-exercise medical evaluation and clearance by a physician before commencing exercise.<br>Referral to trained personnel.

3: Is medical clearance required prior to exercise: □ No □ Yes

If yes, explain why and what action will be taken:

Triage Outcome

Participant triaged to group:

Tick ✓	**Group number**	**Status**	**Exercise prescription**
	1	Meeting recommended physical activity levels	Advice and referral to the PERCS website
	2	Not currently meeting recommended physical activity levels; suitable to exercise without health care professional supervision	Referral to an exercise programme in their local community and to the PERCS website
	3	Not currently meeting recommended physical activity levels and ECOG ≥3, or TUG >13.5 seconds or have comorbidities increasing risk of an exercise-induced adverse event	Referral to a specialist physiotherapy service for individual assessment and treatment.

Document discussion with participant about outcome:

Goal setting:

Set SMART goal(s) with participant for the 12-week programme:

Plan:

e.g. Referral to service, website information, referral to clinician

Action points for participant:

Action points for PERCS team:

Follow up call date: ____________________________

Checklist of measures to be completed at T0:

	**Measure**	**Completed / checked by**	**Date completed / checked**
During subjective ax	Subjective history		
	Patient specific functional scale		
	Mini nutritional assessment		
Physical measures	Height and weight		
	Waist circumference, mid-arm circumference		
	Timed up & go		
	6 minute walking test		
	Hand grip strength test		
	30s sit to stand test		
PROMS	IPAQ		
	Health behaviour & stages of change		
	EORTC-QLQ-C30		
	MFI		
	PHQ-9		
	GAD-7		
Triage	ECOG		
	Pre-participation health screening		
	Triage outcome		

T1 Assessment

Date: / /2023

Participant attended □ Yes □ No
If no, reason for not attending:

Subjective History:

Since T0 assessment:

Any change or update in medical status?

Symptoms and Survivorship Issues

Participants' main problem(s) at present:

Engagement with exercise recommendations:

Participant triaged to level: ☐ 1 ☐ 2 ☐ 3

Was level changed by team at any point: ☐ Yes ☐ No Details:

Recommended exercise plan:

<table>
<tr><td>Level 1: Any change in activity since T0?
☐ Yes
☐ No
Details:</td><td>Level 2/3: Did participant attend service they were referred to?
□ Yes, from T0 – T1
□ Yes, first session only
□ Yes, for other time frame:
□ No, never
□ Other:</td></tr>
<tr><td colspan="2">Weekly diary returned? Yes ☐ No
Completed? ☐ Yes ☐ No
Review diary & gather missing information as able</td></tr>
<tr><td colspan="2">Level 2:
Exercise delivered by which professional:
Describe service:
• Location
• Frequency
• Content
• Cost (direct/indirect)
• Other</td></tr>
<tr><td colspan="2">Level 3:
SJH or other physio:
Date of contact from physio:
Date of first appointment:</td></tr>
</table>

ACSM Recommended Exercise Guidelines (T1)

Aerobic:
"How many days in the past week have you performed physical activity where your heart beats faster and your breathing is harder than normal for 30 minutes or more?"

Days per week engaged in ≥ 30 mins moderate intensity exercise: ___________
Details of exercise: ____

Resistance:
"How many days during the past week have you performed physical activity to increase muscle strength such as lifting weights?"

Days per week engaged in resistance exercise: ___________
Details of exercise: ____

**Participant is meeting the recommended exercise guidelines:**<br>☐ Yes ☐ No<br>(Aerobic ≥ 3 days; resistance ≥ 2 days)

Goal:

Participant's goal (fill from T0 ax):<br><br><br>Participant's reported progress with goal at T1:

Patient Specific Functional Scale

Complete T1 on page 8 of this document ☐

Mini Nutritional Assessment

Sex: ☐ Age: ☐ Weight, kg: ☐ Height, cm: ☐ Date: ☐

Complete the screen by filling in the boxes with the appropriate numbers. Total the numbers for the final screening score.

Screening

A Has food intake declined over the past 3 months due to loss of appetite, digestive problems, chewing or swallowing difficulties?
0 = severe decrease in food intake
1 = moderate decrease in food intake
2 = no decrease in food intake ☐

B Weight loss during the last 3 months
0 = weight loss greater than 3 kg (6.6 lbs)
1 = does not know
2 = weight loss between 1 and 3 kg (2.2 and 6.6 lbs)
3 = no weight loss ☐

C Mobility
0 = bed or chair bound
1 = able to get out of bed / chair but does not go out
2 = goes out ☐

D Has suffered psychological stress or acute disease in the past 3 months?
0 = yes 2 = no ☐

E Neuropsychological problems
0 = severe dementia or depression
1 = mild dementia
2 = no psychological problems ☐

F1 Body Mass Index (BMI) (weight in kg) / (height in m)^2 ☐
0 = BMI less than 19
1 = BMI 19 to less than 21
2 = BMI 21 to less than 23
3 = BMI 23 or greater ☐

IF BMI IS NOT AVAILABLE, REPLACE QUESTION F1 WITH QUESTION F2.
DO NOT ANSWER QUESTION F2 IF QUESTION F1 IS ALREADY COMPLETED.

F2 Calf circumference (CC) in cm
0 = CC less than 31
3 = CC 31 or greater ☐

Screening score ☐☐
(max. 14 points)

12-14 points: ☐ Normal nutritional status
8-11 points: ☐ At risk of malnutrition
0-7 points: ☐ Malnourished

Physical Assessment

Height (cm)		Blood pressure	
Weight (kg)		Heart rate	
BMI (kg/m^2)		SpO2	

Hand Grip Strength

Left	1		Right	1	
	2			2	
	3			3	

**Timed Up and Go**<br>TUG Time: _______ seconds	**30 Second Sit to Stand**<br>30s STS score: _________ stands in 30 seconds

6 Minute Walking Test

Length of course: ______m

Minute	HR	SpO2	Distance<br>*Make a mark for each length walked*
1			
2			
3			
4			
5			
6			Total distance walked:

Observations during physical assessment:

ECOG Status

GRADE	ECOG PERFORMANCE STATUS
0	Fully active, able to carry on all pre-disease performance without restriction
1	Restricted in physically strenuous activity but ambulatory and able to carry out work of a light or sedentary nature, e.g., light house work, office work
2	Ambulatory and capable of all selfcare but unable to carry out any work activities; up and about more than 50% of waking hours
3	Capable of only limited selfcare; confined to bed or chair more than 50% of waking hours
4	Completely disabled; cannot carry on any selfcare; totally confined to bed or chair
5	Dead

Participant ECOG score: __________

Checklist of measures to be completed at T1:

	**Measure**	**Completed / checked by**	**Date completed / checked**
During subjective ax	Subjective history		
	Patient specific functional scale		
	Mini nutritional assessment		
Physical measures	Height and weight		
	Waist circumference, mid-arm circumference		
	Timed up & go		
	6-minute walking test		
	Hand grip strength test		
	30s sit to stand test		
PROMS	IPAQ		
	Health behaviour & stages of change		
	EORTC-QLQ-C30		
	MFI		
	PHQ-9		
	GAD-7		
	Service evaluation		
Other	ECOG		

Incident Tracking

Did an incident occur during the assessments?

☐ No

☐ Yes, a mild incident (such as transient dizziness or headache)

Date: ....

Description (Please include description of event, treatment, attribution and date issue resolved):

☐ Yes, a severe incident(such as chest pain, fainting)

Date: ....

Description (Please include description of event, treatment, attribution and date issue resolved):

Note: In the case of severe incident, inform the coordinating investigator immediately.

☐ Yes, a serious incident occurred (permanent damage or life-threatening situation)

Date: ....

Description (Please include description of event, treatment, attribution and date issue resolved)

Note: In the case of a serious incident, inform the principal investigator immediately.
